# Computed tomography (CT) scanning to visualize eye formation and internal structure in Grevé cheese

**DOI:** 10.1016/j.heliyon.2024.e33408

**Published:** 2024-06-25

**Authors:** Hasitha Priyashantha, Lars Hansson, Peter Forsman, Åse Lundh, Mårten Hetta

**Affiliations:** a Department of Molecular Sciences, Swedish University of Agricultural Sciences, Box 7015, SE-750 07, Uppsala, Sweden; b Department of Engineering Sciences and Mathematics, Luleå University of Technology, Forskargatan 1, 93187, Skellefteå, Sweden; c Department of Ocean Operations and Civil Engineering, Faculty of Engineering, Norwegian University of Science and Technology, 6025, Ålesund, Norway; d Norrmejerier, Mejerivägen 2, SE-906 22, Umeå, Sweden; e Department of Applied Animal Science and Welfare, Swedish University of Agricultural Sciences, SE–901 83, Umeå, Sweden

**Keywords:** Computed tomography (CT) scanning, Grevé cheese, Eye formation, Non-destructive imaging, Cheese quality control

## Abstract

The Grevé cheese, a semi-hard Swedish cheese, is well-known for its characteristic flavor and shape of eye formation. The size and distribution of the eyes play a crucial role for the sensory attributes, aesthetic value and quality of the cheese. This article focuses on investigating the feasibility of using computed tomography (CT) scanning as a non-destructive tool to observe early-stage eye formation in Grevé cheese within an industrial trial. It is crucial to achieve a perfect combination of small and big sized eyes, evenly distributed within the cheese wheel, without having cracks/splits for optimal quality. Such variations could be visualized using CT-scanning of cheeses at a young and mature stage by providing high-resolution, three-dimensional CT-scanning images of the cheese's internal structure, without the need for physical dissection. Further, the distribution of eyes, their shape and number, could be visualized and compared for the same cheese scanned at young and mature stages. Thus, the importance of monitoring eye formation through non-destructive techniques is emphasized to ensure consistent product quality.

## Highlights

• CT scanning assesses Grevé cheese eye formation non-destructively.
• Detects and quantifies cheese eye formation variations effectively.
• Enhances cheese production due to quality control with CT scanning.
• Improves cheese ripening management practices through process optimization.

## 1 Introduction

Computed Tomography (CT) scanning, known for its versatile non-destructive imaging capabilities, has diversified its conventional medical applications to provide valuable insights into various fields, including the assessment of cheese quality [[1], [2], [3]]. While initially developed for medical diagnostics, the power of CT scanning to disclose the internal structure and distribution of physical objects with varying densities as described by Hsieh & Flohr [2], has proven useful in industrial applications in the food science domain. In the context of cheese, CT scanning serves as a powerful and non-destructive tool [4] for understanding the complex interplay of microorganisms, metabolic pathways, and technological factors influencing the formation of the characteristic "cheese eyes" (gas holes) in various cheese varieties, including Emmental, Comté, Jarlsberg, Gouda, and Tilsit, etc [1,5]. These cheese eyes offer more than aesthetic appeal, where they serve as a trademark of the quality and an indicator of cheese structure.

The formation of eyes during cheese maturation and the metabolic pathways responsible for gas production have been subjects of interest over the past decades. Recent attempts have focused on the development of novel eye-forming cultures and further elucidation of the intricate mechanisms that govern cheese eye formation [1,6]. The key players during the ripening process of Swiss-type cheeses are *Propionibacteria* and heterofermentative lactic acid bacteria, which contribute to the formation of CO_2_ [5,7]. Achieving the requisite saturation of the cheese matrix with CO_2_ gas for eye formation demands a careful balance between high CO_2_ production and controlled diffusion [1,8].

The presence of anomalies in the cheese wheels in the form of slits, cracks, and irregularly shaped eyes can lead to the downgrading of cheese quality, thereby necessitating precise and objective quality assessment methods [9]. Traditional methods, often reliant on sensory perception, such as listening to the sound upon tapping the cheese or visual inspection of cheese samples (sample destructive), have their limitations, as they can yield imprecise and non-quantifiable results.

To address these limitations, advanced non-destructive methods have emerged to evaluate the ripening process of cheeses. These encompass digital imaging using cameras and image analysis [10], geometrical measurements [9], ultrasound [11], magnetic resonance imaging (MRI) [12], NIR hyperspectral image modelling [13,14] and CT scanning [1,15]. Among these, CT scanning has demonstrated a remarkable ability in the non-invasive analysis of cheese, particularly in the precise quantification of cheese eye volumes and the evaluation of their spatial distribution. Examples of the application of CT scanning in cheese research are plentiful, e.g. Refs. [1,8,15,16]. These works elucidate how cheese eye characteristics evolve, highlighting the non-destructive potential of CT scanning in tracking the development of cheese eyes. The primary objective of this study was to employ CT scanning for a comprehensive examination of eye development in Grevé, a Swedish cow's milk cheese of the Emmentaler type. We have aimed to assess the feasibility of using CT scanning as a non-destructive tool for studying eye formation in Grevé cheese, enabling quantification of eye volume and its number and qualitative early detection of potential variations. These insights will serve as crucial process control parameters, ultimately contributing to the enhancement and maintenance of cheese quality.

## 2 Materials and methods

### 2.1 Study material and design of the study

**Fig. 1:**
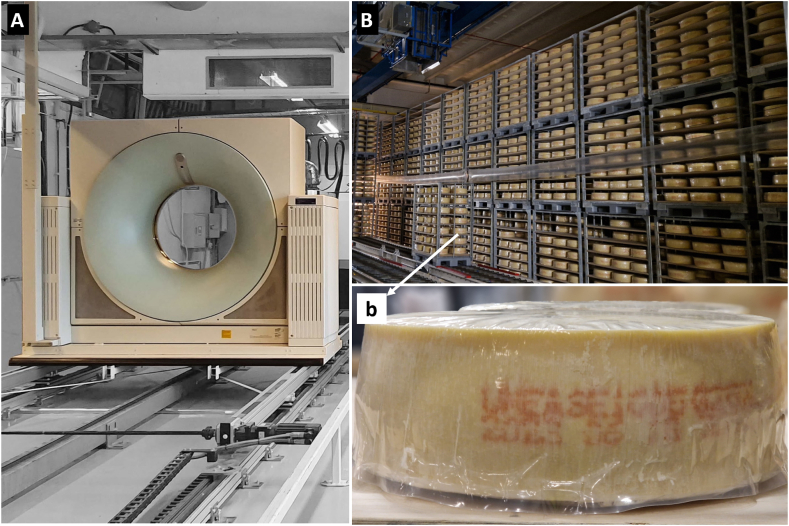
A: The Siemens Somatom Emotion Duo CT scanner (A) was used to scan the Grevé cheeses (b) in a non-destructive manner; the cheese was undisturbed, covered with wax and plastic cover during the scanning process. B: Grevé cheese ripening facility, where cheeses were stored for eye development and in between CT scanning.

This study was conducted based on a full-scale commercial Grevé cheese manufacturing process at Norrmejerier (Umeå, Sweden). The Grevé cheese used as an example in this study (Fig. 1A) is a semi-hard Swedish cheese produced from pasteurised cow milk using mesophilic DL-starter cultures and propionic acid bacteria [17]. In brief, cooking temperatures up to approximately 40 °C were applied according to the commercial cheese-making procedures as specified by the manufacturer. The cheeses are brined and then ripened for two weeks at 9–12 °C. After this initial ripening, the cheeses are covered with a 1 mm layer of wax and ripened for another two weeks at 14–18 °C. Finally, the cheeses are stored for several months at 6–14 °C before being sold. The entire ripening process takes place at the manufacturer's ripening facility. In this designed study, a total of 30 cheeses were utilized, sourced from three distinct production batches, with 10 cheeses from each batch. Two of the batches were crafted on the same day, while the third batch was produced within the same week. The scanning of these cheeses was conducted at two time points: approximately 44 days post-production and around 170 days post-production, corresponding to the mature cheese stage. Maturation took place at the industrial cheese ripening facility (Fig. 1B).

### 2.2 CT scanner configuration

A Siemens Somatom Emotion Duo CT scanner (Fig. 1A), conventionally used for medical imaging, was adapted for the precise analysis of different batches and maturation stages of the Grevé cheese. The CT scanner was equipped with an X-ray tube and four rows of detectors at opposing ends, capable of rotating around a gantry holding the cheese samples during scanning.

### 2.3 Scanning parameters

**Fig. 2:**
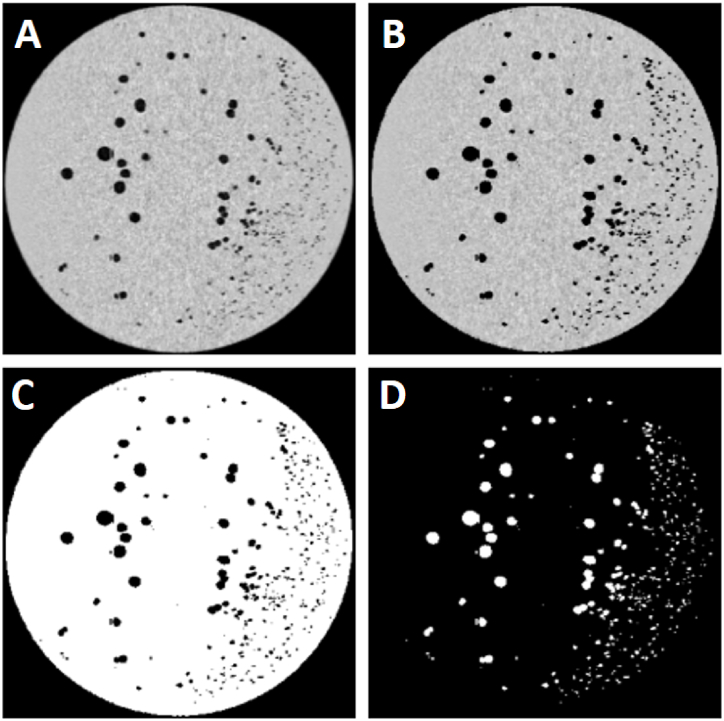
Steps in CT scan processing; (A) Raw image captured from the CT scanner, (B) The same cheese after setting the threshold value of 850 kg/m³, differentiated from the background, (C) Binary image of cheese illustrating cheese features (cavities) within the cheese wheel, (D) Cheese image with the cheese wheel removed, showing only the cavities (eyes).

The scanner was configured with settings of 110 kVp (kilovolt peak) and 88 mAs (milliampere-seconds) for each scanning cycle, resulting in approximately 1000 projections per revolution of the X-ray tube and detectors. The acquired data were transformed into two-dimensional images (Fig. 2A) using a back-projection algorithm as outlined by Hsieh (2009) [18].

### 2.4 Image reconstruction and quality

The resulting images were reconstructed and saved in DICOM (Digital Imaging and Communications in Medicine) format, with a pixel resolution of 1 × 1 mm^2^ and a voxel depth of 1.25 mm. CT imaging method relies on the principle of measuring the extent of X-ray radiation that can penetrate a material, which is determined by the material's attenuation coefficient (Kalender, 2011). The foundational theory of CT is based on Lambert-Beer's law, illustrating that the relationship between the intensity of the transmitted X-ray and the attenuation coefficient is exponential, Formula 1:

(1) $$I=I_{0}e^{-\mu d}$$

Here, *I,* represents the intensity of the X-ray beam after passing through the material, *I*_0_ is the initial intensity of the X-ray beam, μ denotes the linear attenuation coefficient, and *d* is the material's thickness.

The resulting CT images are displayed in shades of grey, with the grey levels closely correlating to the material's density on a nearly linear scale—darker shades indicate lower density, while lighter shades signify higher density.

**Attenuation Coefficient (μ) for Cheese:** The attenuation coefficient μ mentioned is not a predetermined value but rather a result obtained from the CT scanning process. This value is inherently characteristic of the scanned material and is determined by the interaction of X-ray beams with the cheese, thereby being independent of the material type. The observed μ value for cheese reflects its specific density and composition properties under X-ray examination.

**Value of Water Density and Temperature Dependence:** The study utilized a water density value at room temperature. This approach ensures that the measurements are consistent and reliable, irrespective of minor temperature variations.

Given the detailed foundation provided in the literature, our focus shifts to applying CT technology as a quality control tool, uniquely adapted for cheese production and potentially extendable to other industries such as bakery, meat, and chocolate production, where specific settings and adaptations have been developed to meet industry-specific requirements.

### 2.5 CT numbers and density calculation

Each reconstructed image contained CT Numbers (CTN) measured in Hounsfield units. The CTN values were then translated into density measurements in kg/m³. These conversions were based on formula 2, where μ is the linear attenuation coefficient of the cheese material, and $\mu_{w}$ and $\mu_{a}$ are the attenuation coefficients for water and air, respectively.

(2) $$\text{CTN}=1000\;\frac{\mu -\mu_{w}}{\mu_{w}-\mu_{a}}$$

Subsequently, the CTN values were converted to density measurements in kg/m³, following formula 3, where ρ represents the density of the cheese sample being examined.

(3) ρ=CTN–1024

### 2.6 Software utilization

The software utilized for the study was 3D Slicer, an open-source platform known for its versatility in medical image informatics and image-based research. The software was configured for the specific purpose of analysing the internal structure of the cheese wheels using CT scans as described below.

### 2.7 Image thresholding

To delineate cheese regions in CT images, image thresholding techniques were employed to isolate and analyze the cheeses within complex internal structures. The process of setting a threshold value is crucial for accurately distinguishing different materials based on their densities. In this study, the threshold value was set manually at 850 kg/m³ based on the typical density range of cheese, which is approximately 1100–1200 kg/m³. This threshold effectively differentiates the cheese from the surrounding background in the DICOM image data, as materials with densities below this threshold appear distinctly different from cheese (Fig. 2B).

The manual threshold selection approach was adopted because it can provide greater control over the segmentation process when the material properties are well-known and consistent. While useful in scenarios with variable or unknown material characteristics, automatic thresholding methods can sometimes lead to suboptimal segmentation due to their reliance on generalised statistical information from image histograms. By manually setting the threshold, it is ensured that the specific characteristics of cheese, such as its uniform density and distinct textural properties, are accurately captured in the CT scans. This method allows for precise segmentation of cheese regions, which is critical for the subsequent analyses of structure and quality. This manual approach was implemented within 3D Slicer, facilitating the separation of cheese regions from the background in the CT scans, and ensuring that the image processing was tailored specifically to the unique properties of the cheese.

### 2.8 Cavity analysis

Once thresholding was applied, the binary images (Fig. 2C and D) enabled detailed analysis of cheese features. These analyses included the determination of size, identification, and counting of cavities or eyes, and shape analysis to assess the roundness of these cavities using advanced image analysis techniques. These quantitative measures provided valuable insights into the cheese's internal structure, texture, and quality.

#### 2.8.1 Binary image processing

The binary images (2C and 2D) result from applying a thresholding technique to the original CT images. This step converts the grayscale images into a binary format, where the cavities (eyes) and cheese matrix are distinctly separated into binary values (0 for cavities and 1 for cheese matrix), facilitating the identification and analysis of cavities.

#### 2.8.2 Identification of cavities

Using the binary images, individual cavities are identified through image segmentation techniques. These techniques group contiguous regions of '0's (representing cavities) within the binary images, enabling the isolation and examination of each cavity.

#### 2.8.3 Volume calculation from 2D to 3D projection

Although the binary images are two-dimensional, the volume of each identified cavity is calculated by extrapolating the 2D data into a 3D representation. This extrapolation is based on the slice thickness of the CT scans and the known dimensions of each pixel within the image. Specifically, the area of each cavity in the binary images is calculated and then multiplied by the slice thickness to estimate the volume of each cavity in three-dimensional space.

#### 2.8.4 Conversion to cubic centimeters

The calculated volumes, initially in pixel³ units, are converted into cubic centimetres (cm³) using the calibration factors of the CT scanner, which include the physical dimensions of each pixel and the slice thickness. This conversion allows for the expression of eye size in a standardised and easily interpretable unit of measure. This methodology ensures that the eye size, expressed as a volume in cm³, is calculated accurately from the binary images. The combination of advanced image processing techniques and precise calibration of the CT scan parameters facilitates the detailed analysis of the internal structure of the cheese, including the size and distribution of cavities.

### 2.9 Data analysis

Statistical analysis of cheese eye characteristics, calculated using CT images, was conducted using one-way analysis of variance (ANOVA) in Minitab 18.1 software (Minitab Inc., State College, PA, USA). Subsequently, a Tukey post-hoc test was employed for pairwise comparisons. Significance was determined at a threshold of P < 0.05.

## 3 Results and discussion

**Fig. 3:**
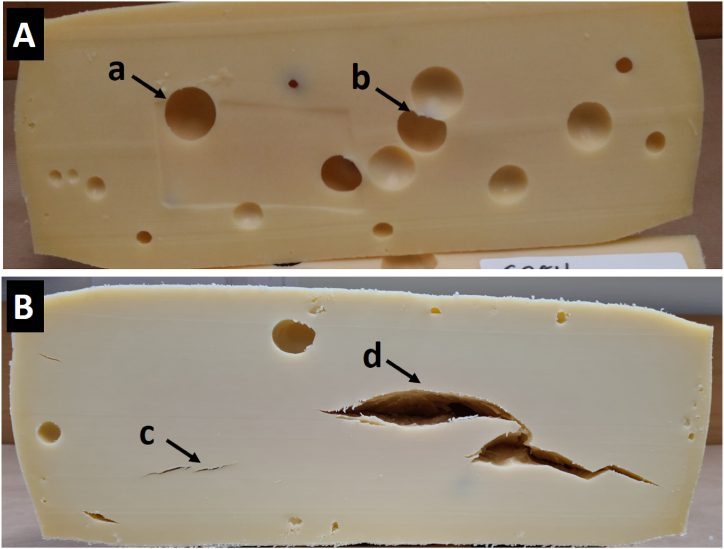
A: Grevé cheese cross-section illustrating a perfect distribution of both small and big eyes, with a round single eye (a) and coalescence of two eyes (b). B: Grevé cheese cross-section with almost invisible (c) and distinct (d) cracks/splits.

The Grevé cheese is a well-known and appreciated semi-hard cheese with long tradition in the Swedish dairy industry, characterized by its distinct eyes resulting from gas production during fermentation [17]. Thus, the development of eyes in the Grevé cheese and the detection of defects are of great interest since they significantly impact its texture, flavour, and overall quality. Moreover, this has huge impact on the market price for the producer, since it is not allowed to sell as a Grevé cheese with defected texture. Achieving an ideal combination of small and big eyes (Fig. 3A) is essential for creating the desired sensory attributes and ensuring consumer satisfaction. Cracks, slits, or splits developing inside the cheese wheel (Fig. 3B) diminish its aesthetic appeal and marketability. These defects typically emerge during the advanced stages of cheese ripening, becoming visible as cracks or as the tearing off of existing eyes. Guggisberg et al. (2015) [1] explained this defect by an insufficient eye number and thus increased CO_2_ overpressure within the cheese body. Thus, considerable amount of flexibility in the cheese body is essential to form nice round eyes and in case of the too hard and brittle cheese body, the pressure from the gas will cause splits and cracks in the cheese body.

While cheese producers must strive to minimize the occurrence of eye defects through meticulous production practices, proper ripening and storage conditions, and rigorous quality control measures are of great importance. Moreover Guggisberg et al. (2015) [1] reviewed factors such as acidification (leading to the solubilisation of calcium), moulding and pressing (influencing technical openness), proteolysis (which reduces the elasticity of the cheese body), low or sudden changes in storage temperatures (affecting the plasticity of the cheese body), all playing significant roles. However, producers need to consider and address all relevant factors to maintain the integrity and quality of their cheese products throughout the production and ripening process. Thus, the use of CT scanning could be an important non-destructive tool to control eye characteristics and detect defects already at an early stage, as previously described by Kraggerud et al. (2009) [4].

**Fig. 4:**
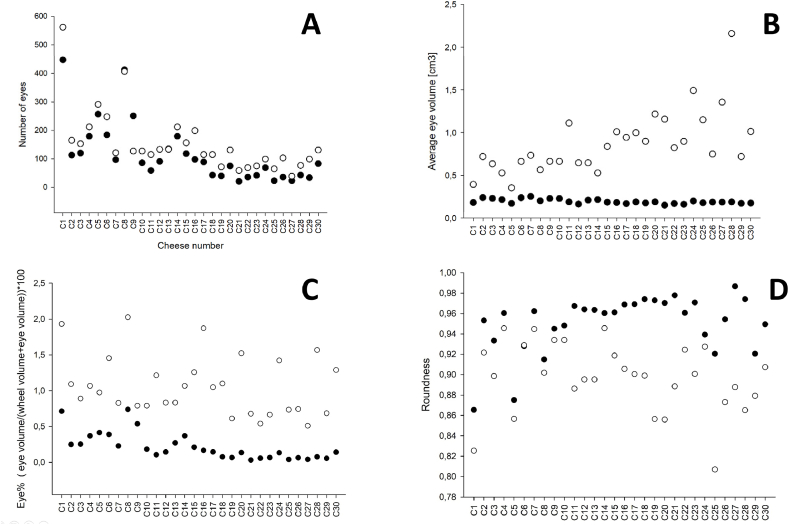
Individual variation of (A) Number of eyes, (B) Average eye volume, (C) Eye% = eye volume/(wheel volume + eye volume)*100 and (D) Roudness of the eyes in studied cheese at young (44 days): dark symbols and mature (170 days): light symbols. Add info about cheese batches.

**Fig. 5:**
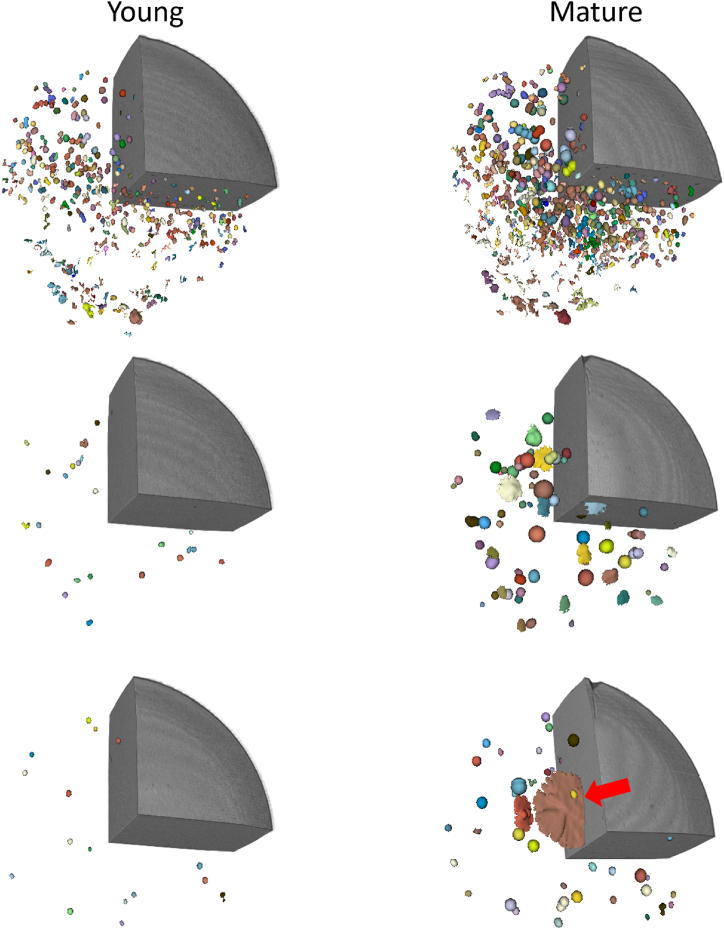
CT-scan images representing the progression of experimental cheeses, highlighting their unique attributes at both the early (44 days; illustrated on the left) and mature (170 days; illustrated on the right) stages. The virtual quarter sections of the cheese wheels have been methodically visualized, enabling a clear representation of eye distribution within the cheese wheel. An arrow indicates a crack or split in a particular cheese.

Table 1 provides a comparison of younger and mature Grevé cheeses’ eye characteristics. Both young and mature cheeses had a similar average number of eyes per wheel, with no significant difference observed. Probably, this could be due to the relatively high variation between the cheeses (Fig. 4 A). Another probable reason for not observing the higher number of eyes in young cheese could be the established image threshold value at 850 kg/m^3^, allowing the cheese to be effectively distinguished from the background. In general, young cheeses exhibit a greater abundance of eyes attributed to increased starter culture activity during its early production stages. Lactose, the milk sugar, serves as the substrate for the metabolic processes of the starter cultures, resulting in the production of lactic acid. Subsequently, certain strains of *Propionibacterium* further metabolize the lactic acid, yielding carbon dioxide (CO_2_) by forming gas holes, acetic acid, and propionic acid. Additionally, the higher moisture content in an early stage of ripening provides a conducive environment for gas formation compared to the latter stages [[19], [20], [21]]. The average volume of individual eyes was significantly larger in the mature cheeses than in the young cheeses (Table 1), indicating further growth and development of the eyes as the cheese matured (Fig. 4B). The mature cheeses exhibited a significantly higher total eye volume compared to the young cheeses in agreement with Fröhlich-Wyder & Bachmann (2004) [5], who reported that eye enlargement occurs after 50 days of postproduction in Swiss-type cheeses. Thus, when the cheese matures, the size of the eyes increases substantially as shown in Fig. 4B and visualized in Fig. 5.

**Table 1:** Comparison of CT-scan cheeses at young and mature stages.

Characteristic	Young cheese	Mature cheese
Age (days)	44	170
Number of Eyes	116.20 ± 106.40^a^	153.70 ± 108.40^a^
Average eye volume (cm^3^)	0.19 ± 0.02^b^	0.87 ± 0.37^a^
Average eye voume 10th percentile (cm^3^)	0.10 ± 0.01	0.15 ± 0.01
Average eye voume 90th percentile (cm^3^)	0.31 ± 0.05	1.89 ± 0. 77
Total eye volume (cm^3^)	23.09 ± 21.11^b^	113.43 ± 47.09^a^
Ratio	0.002 ± 0.001^b^	0.010 ± 0.004^a^
Eye%	0.21 ± 0.19^b^	1.07 ± 0.41^a^
Roundness of the eye	0.95 ± 0.03^b^	0.89 ± 0.03^a^
Average eye roundness 10th percentile	0.82 ± 0.08	0.65 ± 0.09
Average eye roundness 90th percentile	1.01 ± 0.01	1.00 ± 0.01

The ratio of eye volume to total cheese volume was significantly higher in mature cheeses compared to young cheeses (Table 1), indicating a greater proportion of eyes relative to the overall cheese structure in mature cheeses. Moreover, the percentage of eye volume relative to the total cheese wheel volume including the eyes and cheese body was significantly higher (Table 1) in mature cheeses compared to young cheeses (Fig. 4C), indicating a higher eye volume density in the mature cheese. This may probably also be influenced by the presence of cracks in mature cheeses compared to young cheeses. Similarly, the roundness of individual eyes was significantly lower (Table 1) in mature cheeses compared to young cheeses (Fig. 4D), suggesting that the eyes in mature cheeses may be more irregular or asymmetrical in their shapes (Fig. 5), probably due to the aforementioned reason. Thus, CT scanning allows for precise measurement and quantification of various parameters related to eye formation as discussed above and depicted in Table 1. This possibility of having quantitative data provide also future possibilities in assessing the impact of different processing variables (e.g., milk composition, cheese-making techniques, ripening conditions) on eye formation and product quality, providing valuable insights for process optimization and quality control.

Ratio = eye volume/wheel volume, Eye% = eye volume/(wheel volume + eye volume)*100. Note: Wheel volume is just consisting of the protein/fat network, thus not including the volume of the eyes. Roundness = the geometric characteristic used to evaluate the shape of the eye, ranging from 0; less round to 1; more round. Calculated roundness values can sometimes exceed 1.0 due to irregular shapes of eyes or bigger cracks. The value ± standard deviation is indicated.

Hence using non-destructive techniques such as CT scanning to study eye formation in Grevé cheese offers several significant advantages and insights into the cheese ripening process and product quality. CT scanning provides high-resolution, three-dimensional images of the internal structure of cheeses (Fig. 5), allowing cheese manufacturers to visualize the distribution, size, and morphology of eyes without the need for physical dissection. This enables a detailed examination of the cheese's internal characteristics, including the arrangement of protein and fat matrices (macroscopic overview of the cheese wheel), gas distribution, and the presence of defects such as cracks or splits (Fig. 5). One major advantage is that these defects can be detected at an early stage, so that defected cheese or cheese batches can be removed and processed into grated cheese or perform another marketing strategies.

The CT scanning facilitates process optimization and quality assurance of Swiss-type cheeses, e.g. Grevé cheese production by enabling real-time, non-destructive monitoring of eye formation during cheese production and ripening as described in this paper. This further provides the opportunity to use CT scans to track changes in eye formation over the time as an online process quality control tool, identify potential issues or irregularities, and make timely adjustments to production parameters to achieve desired product characteristics and consistency in cheese production. Therefore, CT scanning can serve as a valuable tool for quality control and assurance in cheese production, allowing producers to detect and assess internal defects or abnormalities that may impact the product quality of e.g. Grevé cheese. By identifying issues such as uneven eye distribution, excessive gas formation, or structural irregularities, CT scanning helps to ensure that only cheeses meeting quality standards are released to the market, thereby enhancing consumer satisfaction and brand reputation. However, it's important to highlight that CT scanners are hi-tech and costly, rendering them inaccessible to many industries due to their high investment requirements. Hence it would be worth exploring the potential of utilizing more affordable and food industry-oriented CT scanners to achieve similar outcomes. We also suggest that CT scanning has a wide potential to contribute to ongoing research and innovation in cheese-making by providing researchers with a powerful tool for studying the underlying mechanisms and dynamics of eye formation. By gaining a deeper understanding of the factors influencing eye development, researchers may explore new approaches, technologies, and interventions to manipulate and optimize eye formation in cheese at a time of increasing diversification of on-farm factors, milk quality attributes and processing conditions associated with the dairy value chain.

## 4 Conclusions

The CT scanning methodology presented in this paper, which encompasses image thresholding and subsequent analyses, represents a significant contribution to the field of cheese quality analysis. This comprehensive approach allows for examining the internal structure of Grevé cheese and similar varieties with unique detail and accuracy. By employing this methodology, we have demonstrated its utility in providing both quantitative and qualitative data essential for various quality control applications. The quantitative data obtained through CT scanning is invaluable for quality control purposes, aiding in assessing cheese texture attributes and ensuring consistency in production processes. Additionally, the qualitative insights gained from this method enhance our understanding of cheese eye formation and other structural characteristics. Moreover, the non-destructive nature of CT scanning enables real-time process monitoring and quality control in cheese production settings. This capability has significant implications for the food industry, allowing producers to identify and address issues promptly, thereby enhancing overall product quality and consumer satisfaction. We believe that our findings will contribute to improving quality control practices and deepen the understanding of cheese production processes, ultimately benefiting both producers and consumers. Future work may entail the exploration of CT technology application in diverse cheese varieties, optimization of imaging parameters for improved accuracy, integration of advanced analytics such as machine learning, collaboration with industry stakeholders for real-world implementation, and conducting longitudinal studies to monitor quality variations over time.

## Ethical statement-studies in humans and animals

There are no human subjects in this article and informed consent is no applicable.

## Ethical statement

This work does not involve trials on any human or animals.

## Funding

This study was funded by Norrlandsnavet a center for business development in northern Sweden in collaboration with the 10.13039/501100009750Kamprad Family Foundation and Luleå University of Technology and the Regional 10.13039/501100004379Foundation for Agricultural Research in Northern Sweden (Regional jordbruksforskning för norra Sverige; grant **RJN September 2021.**

## Data availability statement

The original contributions presented in the study are included in the article. further inquiries can be directed to the corresponding author.

## CRediT authorship contribution statement

**Hasitha Priyashantha:** Writing – review & editing, Writing – original draft, Visualization, Validation, Software, Investigation, Formal analysis, Data curation. **Lars Hansson:** Writing – review & editing, Validation, Software, Resources, Methodology, Investigation, Formal analysis, Data curation. **Peter Forsman:** Writing – review & editing, Resources, Investigation, Formal analysis. **Åse Lundh:** Writing – review & editing, Validation, Supervision, Resources, Project administration, Funding acquisition, Conceptualization. **Mårten Hetta:** Writing – review & editing, Validation, Supervision, Resources, Project administration, Methodology, Investigation, Funding acquisition, Data curation, Conceptualization.

## Declaration of competing interest

The author declares that the article was complied with in the absence of any commercial or financial relationships that could be construed as a potential conflict of interest.
